# Supplementary material for: Gut Microbiota and Associated Mucosal Immune Response in Eosinophilic Granulomatosis with Polyangiitis (EGPA)
Source: Biomedicines. 2022 May 24;10(6):1227. doi: 10.3390/biomedicines10061227 (PMC9219964; doi:10.3390/biomedicines10061227)
Supplement: Supplementary file 1 [file biomedicines-10-01227-s001.zip › biomedicines-1696416-supplementary.pdf]

SUPPLEMENTARY FIGURE

Figure S1. Rarefaction curves showing the level of saturation of amplicon sequence variants (ASVs).

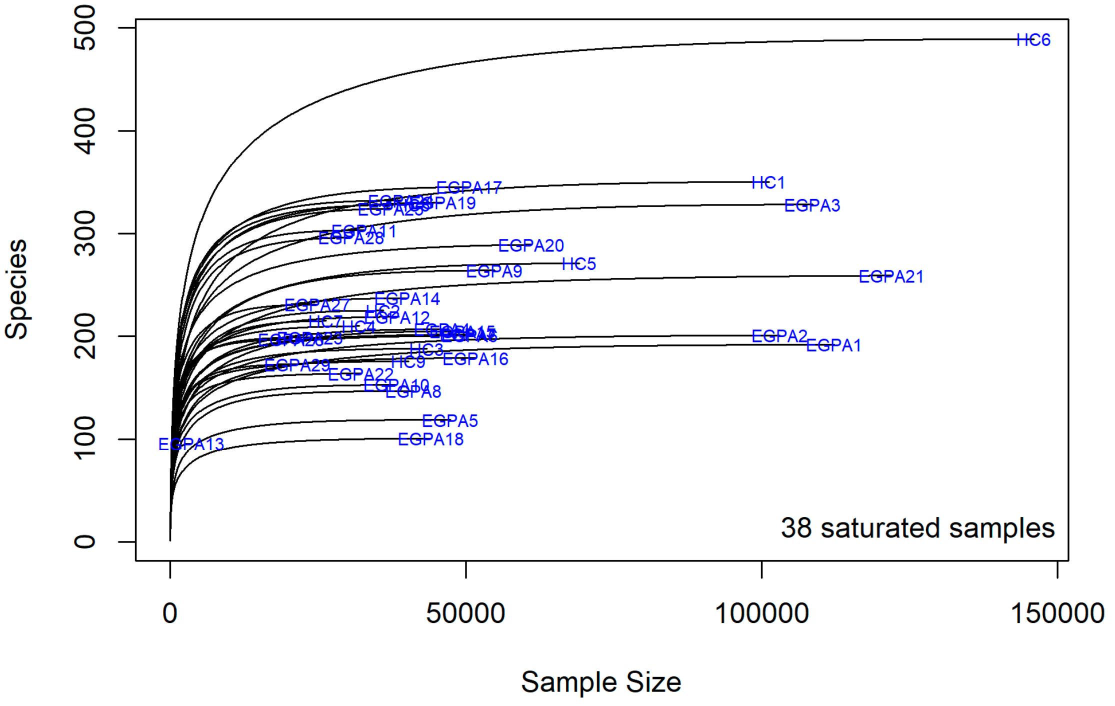

Figure S2. Significantly different levels of gut microbiome-derived free fatty acids among patients with distinct clinical features. GI= gastrointestinal involvement; Eos= Eosinophilia.

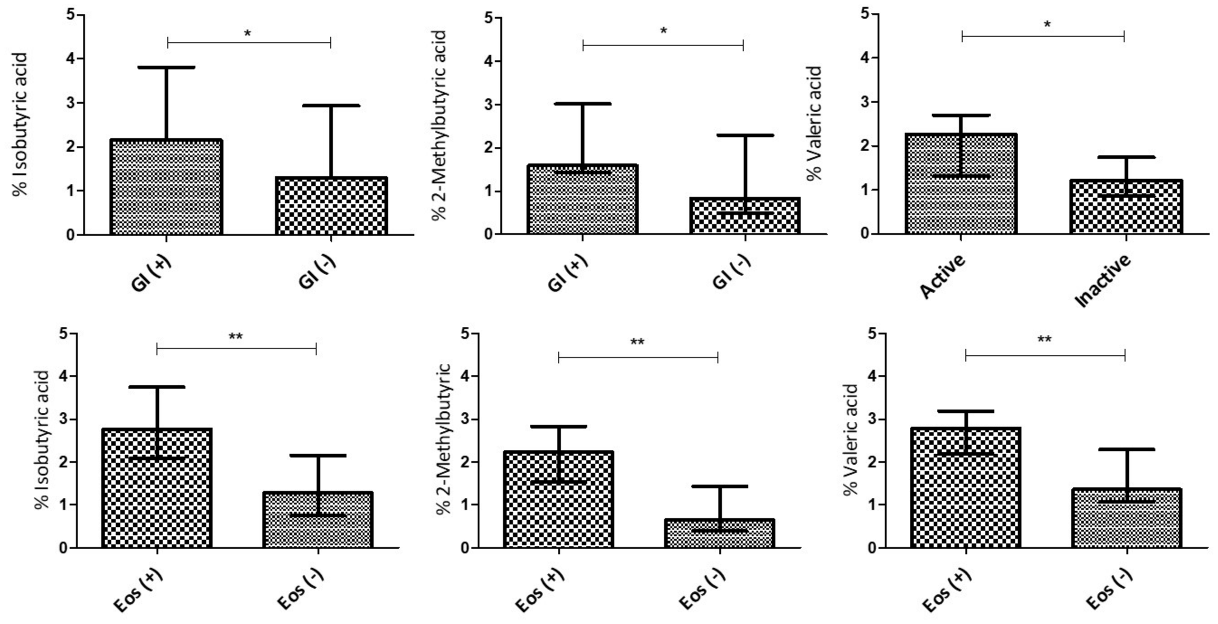

UPPLEMENTARY TABLES

Table S1. Gut microbiome-derived free fatty acids obtained from stool of EGPA patients and healthy controls. p-value assessed with Mann-Whitney test.

**SCFA and MCFA**	**EGPA**	**Healthy controls**	**p value**
**Total free fatty acids** (median (IQR) µmol/g)	42.61 (27.92)	51.05 (30.45)	0.16
**Percentage of each fatty acid** (median (IQR);%)			
Acetic acid	67.62 (9.22)	67.33 (19.61)	0.275
Propionic acid	14.22 (5.82)	12.82 (5.43)	0.737
Butyric acid	11.14 (6.51)	11.05 (2.28)	0.777
isoButyric acid	1.75 (1.63)	1.45 (1.87)	0.873
isoValeric acid	1.21 (1.69)	1.01 (1.25)	0.777
2-MethylButyric acid	1.17 (1.63)	0.89 (1.27)	0.901
Valeric acid	2.06 (1.11)	2.27 (1.20)	0.671
Hexanoic acid	0.00 (0.59)	0.62 (0.72)	0.015

Table S2. Correlations between T cell count, patients' clinical features, and mucosal bacterial composition from colonoscopy biopsies from EGPA patients.
Correlations are reported as Spearman's Rho and p-values.

	**Phyla**				
	**Bacteroidota**	**Proteobacteria**	**Firmicutes**	**Fusobacteriota**	**Actinobacteriota**
**Tot n of clones**	0.964; p=0.000*	0.214; p=0.644	-0.60; p=0.148	-0.07; p=0.866	0.464; p=0.293
**Th1**	-0.53; p=0.215	-0.64; p=0.119	0.642; p=0.119	-0.78; p=0.035*	-0.21; p=0.644
**Th1/Th17**	0.428; p=0.337	0.392; p=0.383	-0.57; p=0.180	0.216; p=0.640	0.214; p=0.644
**Th17**	-0.17; p=0.701	0.25; p=0.588	0; p=1	0.453; p=0.307	0.142; p=0.759
**Th0**	0.481; p=0.273	-0.25; p=0.574	-0.03; p=0.937	-0.65; p=0.110	0.296; p=0.518
**Th2**	0.118; p=0.800	-0.43; p=0.331	0.197; p=0.671	-0.68; p=0.089	-0.15; p=0.735
**Treg**	-0.17; p=0.701	0.392; p=0.383	-0.25; p=0.588	0.866; p=0.011*	-0.39; p=0.383
**Tc1**	0.357; p=0.431	0.357; p=0.431	-0.28; p=0.534	0.157; p=0.735	0.428; p=0.337
**Tc1/Tc17**	0.481; p=0.273	0.704; p=0.077	-0.70; p=0.077	0.899; p=0.005*	-0.07; p=0.874
**Tc17**	0.216; p=0.640	0.315; p=0.491	-0.47; p=0.283	0.380; p=0.399	0.433; p=0.331
**Eosinophil count**	0.821; p=0.023*	0; p=1	-0.46; p=0.293	-0.03; p=0.933	0.392; p=0.383
**BVAS**	0.774; p=0.040*	0.018; p=0.969	-0.41; p=0.355	-0.37; p=0.403	0.432; p=0.332
	**Family**				
	**Bacteroidaceae**	**Enterobacteriaceae**	**Lachnospiraceae**	**Pseudomonadaceae**	**Ruminococcaceae**
**Tot n of clones**	0.785; p=0.036*	0.214; p=0.644	-0.82; p=0.023*	0.25; p=0.588	-0.28; p=0.534
**Th1**	-0.67; p=0.093	0.107; p=0.819	0.5; p=0.253	-0.28; p=0.534	0.107; p=0.819
**Th1/Th17**	0.642; p=0.119	0.464; p=0.293	-0.42; p=0.337	0; p=1	-0.32; p=0.482
**Th17**	0.178; p=0.701	0.071; p=0.879	0.142; p=0.759	-0.14; p=0.759	0.142; p=0.759
**Th0**	0.185; p=0.690	0.148; p=0.751	-0.40; p=0.363	0.148; p=0.751	-0.14; p=0.751
**Th2**	-0.23; p=0.609	0.157; p=0.735	-0.07; p=0.866	-0.15; p=0.735	-0.15; p=0.735
**Treg**	-0.07; p=0.879	-0.35; p=0.431	0.178; p=0.701	0.035; p=0.939	0.071; p=0.879
**Tc1**	0.428; p=0.337	-0.17; p=0.701	-0.42; p=0.337	0.571; p=0.180	-0.03; p=0.939
**Tc1/Tc17**	0.630; p=0.129	0.037; p=0.937	-0.48; p=0.273	0.074; p=0.874	-0.25; p=0.574
**Tc17**	0.256; p=0.579	-0.57; p=0.180	-0.11; p=0.800	0.866; p=0.011*	0.216; p=0.640
**Eosinophil count**	0.642; p=0.119	0.25; p=0.588	-0.53; p=0.215	-0.10; p=0.819	-0.10; p=0.819
**BVAS**	0.522; p=0.228	0.126; p=0.787	-0.66; p=0.101	0.360; p=0.427	-0.23; p=0.613
	**Genus**				

	Bacteroides	Escherichia-Shigella	Anaerostipes	Pseudomonas	[Ruminococcus]_gnavus_group
**Tot n of clones**	0,785; p=0,036	0,214; p=0,644	-0,53; p=0,215	0,25; p=0,588	-0,17; p=0,701
**Th1**	-0,67; p=0,093	0,107; p=0,819	0; p=1	-0,28; p=0,534	0,535; p=0,215
**Th1/Th17**	0,642; p=0,119	0,464; p=0,293	-0,32; p=0,482	0; p=1	0,178; p=0,701
**Th17**	0,178; p=0,701	0,071; p=0,879	0,464; p=0,293	-0,14; p=0,759	-0,07; p=0,879
**Th0**	0,185; p=0,690	0,148; p=0,751	-0,40; p=0,363	0,148; p=0,751	0; p=1
**Th2**	-0,23; p=0,609	0,157; p=0,735	-0,47; p=0,283	-0,15; p=0,735	0,394; p=0,381
**Treg**	-0,07; p=0,879	-0,35; p=0,431	0,142; p=0,759	0,035; p=0,939	-0,17; p=0,701
**Tc1**	0,428; p=0,337	-0,17; p=0,701	0,142; p=0,759	0,571; p=0,180	-0,82; p=0,023
**Tc1/Tc17**	0,630; p=0,129	0,037; p=0,937	-0,18; p=0,690	0,074; p=0,874	-0,25; p=0,574
**Tc17**	0,256; p=0,579	-0,57; p=0,180	0,157; p=0,735	0,866; p=0,011	-0,80; p=0,027
**Eosinophil count**	0,642; p=0,119	0,25; p=0,588	-0,46; p=0,293	-0,10; p=0,819	0,285; p=0,534
**BVAS**	0,522; p=0,228	0,126; p=0,787	-0,52; p=0,228	0,360; p=0,427	-0,19; p=0,670
